# Comment on “Prognostic impact of adjuvant therapy following surgical resection in primary hepatic sarcomatoid carcinoma*”*

**DOI:** 10.1097/JS9.0000000000003390

**Published:** 2025-09-08

**Authors:** Qun Xiang, Xianghui Fu, Xuyang Sun

**Affiliations:** a Department of Infectious Diseases, Hainan Public Health Clinical Center, Haikou, Hainan Province, China; b Department of Gastrointestinal Surgery, Hainan Affiliated Hospital of Hainan Medical University, Haikou, Hainan Province, China; c Department of Interventional Radiology, Hainan Hospital of the General Hospital of the People’s Liberation Army of China, Sanya, China

**HIGHLIGHTS**

- Identifies a gap in exploring molecular markers, such as PD-L1, affecting treatment response.
- Suggests integrating molecular profiling to improve understanding of patient response variability.
- Recommends assessing biomarkers for better patient selection in immunotherapy.
- Calls for further studies to enhance treatment strategies for PHSC through molecular analysis.

*Dear Editor,*

We read with great interest the article titled “Prognostic Impact of Adjuvant Therapy Following Surgical Resection in Primary Hepatic Sarcomatoid Carcinoma”^[1]^. This study offers valuable insights into the role of postoperative adjuvant therapy in improving disease-free survival in patients with primary hepatic sarcomatoid carcinoma (PHSC), and we commend the authors for their significant contribution to the field. However, we would like to address one aspect that may impact the completeness of the findings. This correspondence was developed following the TITAN Guidelines 2025^[2]^ for the declaration and use of artificial intelligence in scholarly publishing. No AI tools were employed in the conception, design, analysis, interpretation, or writing of this work.

While the study provides essential clinical outcomes, it does not explore molecular markers that may influence treatment response, such as PD-L1 expression. Recent studies have shown that PD-L1 expression can play a crucial role in the effectiveness of immunotherapy, particularly in cancers like hepatocellular carcinoma and its variants^[3]^. By incorporating molecular profiling into future studies, the authors could gain a deeper understanding of why certain patients respond better to adjuvant therapy, which would be valuable for refining treatment strategies. This molecular analysis would also help identify biomarkers for predicting which patients would benefit most from immunotherapy and other adjuvant treatments, ultimately improving patient selection.

We believe that addressing this point in future studies could provide a more comprehensive understanding of the treatment strategies for PHSC patients.

## Data Availability

The letter does not have any data.

## Research registration unique identifying number (UIN)

Not registered for the type of study.
